# Transesterification of Glycerol to Glycerol Carbonate over Mg-Zr Composite Oxide Prepared by Hydrothermal Process

**DOI:** 10.3390/nano12121972

**Published:** 2022-06-08

**Authors:** Yihao Li, Hepan Zhao, Wei Xue, Fang Li, Zhimiao Wang

**Affiliations:** Key Laboratory of Green Chemical Technology and High Efficient Energy Saving of Hebei Province, Tianjin Key Laboratory of Chemical Process Safety, School of Chemical Engineering, Hebei University of Technology, Tianjin 300401, China; 201921503002@stu.hebut.edu.cn (Y.L.); hgdzhao16029@hebut.edu.cn (H.Z.); weixue@hebut.edu.cn (W.X.)

**Keywords:** glycerol carbonate, transesterification, hydrothermal process, Mg-Zr oxides

## Abstract

A series of Mg-Zr composite oxide catalysts prepared by the hydrothermal process were used for the transesterification of glycerol (GL) with dimethyl carbonate (DMC) to produce glycerol carbonate (GC). The effects of the preparation method (co-precipitation, hydrothermal process) and Mg/Zr ratio on the catalytic performance were systematically investigated, and the deactivation of the catalyst was also explored. The Mg-Zr composite oxide catalysts were characterized by XRD, TEM, TPD, N_2_ adsorption-desorption, and XPS. The characterization results showed that compared with the co-precipitation process, the catalyst prepared by the hydrothermal process has a larger specific surface area, smaller grain size, and higher dispersion. Mg1Zr2-HT catalyst calcined at 600 °C in a nitrogen atmosphere exhibited the best catalytic performance. Under the conditions of reaction time of 90 min, reaction temperature of 90 °C, catalyst dosage of 3 wt% of GL, and GL/DMC molar ratio of 1/5, the GL conversion was 99% with 96.1% GC selectivity, and the yield of GC was 74.5% when it was reused for the fourth time.

## 1. Introduction

With the increasing consumption of fossil fuels and the consequent environmental problems, especially the threat of global warming, China has put forward the strategic goal of “carbon peaking and carbon neutralization”. The realization of this goal needs to accelerate clean energy substitution and energy transformation. Biodiesel is a promising clean and renewable energy, and has become a hot spot for the sustainable development of global energy and the environment [1]. Biodiesel is obtained by transesterification of vegetable oil and waste oil with methanol or ethanol and will produce by-product glycerol (GL). By 2024, the global biofuel market is expected to reach US $153.8 billion, but for every 1000 kg biodiesel produced, there will be 100 kg GL [2].

In order to solve the problem of crude glycerol utilization, scientists have explored and developed different synthetic procedures to convert GL into high value-added derivatives, such as the steam reforming of glycerol [3,4,5,6,7], catalytic esterification of glycerol, catalytic hydrogenolysis of glycerol and among others. Among various GL derivatives, glycerol carbonate (4-hydroxymethyl-1,3-dioxolane-2-one, GC) has the advantages of low flammability, low toxicity, high boiling point, and biodegradability [8]. It is widely used as a solvent in the cosmetics industry, and can also be used in the manufacture of paint, fiber, plastic, coating, cement curing agent, biological lubricant, and so on [9].

**Scheme 1:**
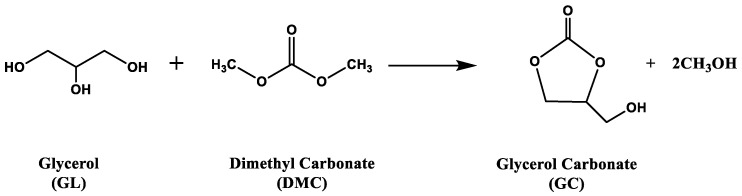
Synthesis of GC by transesterification from GL and DMC.

At present, the routes for the synthesis of GC using GL as raw material mainly include phosgenaion [10], oxidative carbonylation [11], urea alcoholysis [12], and transesterification [13]. Among them, transesterification (Scheme 1) has the advantages of mild reaction conditions and simple operation, which is considered to be one of the most direct and feasible ways in the industry [14].

In recent years, alkali catalysts such as MgO [15,16] and CaO [17] have been widely used in GC synthesis by transesterification. However, CaO can be dissolved into the reactant GL and form a calcium-glycerin bond [18]. Moreover, CaO may produce CaCO_3_ with DMC in the presence of water [19], which reduces the catalytic activity, limiting the reuse of catalysts. In addition, single metal oxides such as MgO and CaO will react with water and CO_2_ in the air during preparation and storage, and then deactivate [20]. In general, composite oxides have stronger acidity and basicity and larger specific surface areas than single metal oxides; the lattice structure can also be changed by doping metal cations with different electronegativity, thereby changing the acidity and basicity of the catalyst surface [21]. So it shows good application prospects in heterogeneous alkali catalytic reactions [22]. Zhang [23] prepared a large specific surface area CaO-ZrO_2_ catalyst with the mesoporous structure for continuous transesterification synthesis of GC in a fixed bed reactor. Under the optimized conditions, the yield of GC can reach 90%. However, the catalysts prepared by the co-precipitation process have some disadvantages, such as easy loss of active components and deactivation due to carbon species deposited on the surface [24]. The hydrothermal process has been widely used in the synthesis of oxide nanoparticles in recent years. Compared with other preparation processes, hydrothermally synthesized nanoparticles have high purity, good dispersibility, and controllable grain size [25]. Cui prepared MgO nanosheets with a two-dimensional flaky porous structure by simple hydrothermal process, which has a larger specific surface area than commercial MgO nanoparticles [26]. The ZrO_2_ nanocrystals prepared by hydrothermal synthesis of Akune [27] show high catalytic activity due to their high specific surface area and high crystallinity. Wang compared the Mg/Sn/W composite oxide catalysts prepared by co-precipitation process and hydrothermal process, and pointed out that the catalysts prepared by the hydrothermal process had smaller particles, higher thermal stability, and catalytic activity [28].

In this article, Mg-Zr composite oxide catalysts with different Mg/Zr molar ratios were prepared by hydrothermal process for the transesterification of GL and DMC to synthesize GC. The effects of the preparation method and Mg/Zr molar ratio were systematically investigated. The catalysts were characterized by XRD, N_2_ adsorption-desorption, TEM, TPD, and XPS, and the structure-activity relationship of the Mg-Zr oxide catalysts was discussed. In addition, the transesterification reaction conditions were optimized, the reusability of the catalyst was investigated, and the deactivation reasons of the catalysts were explored.

## 2. Materials and Methods

### 2.1. Materials

Glycerol (99%, AR); methanol (99.5%, AR); magnesium nitrate hexahydrate (99%, AR) and sodium hydroxide were purchased from Kemio Reagent Co, Ltd. (Tianjin, China). Zirconium oxychloride octahydrate (99%, AR); dimethyl carbonate (99%, AR), and n-butanol (99%, AR) were purchased from Damao Chemical Reagent Factory (Tianjin, China). Glycidyl (98%) and glycerol carbonate (90%) were purchased from Aladdin Biochemical Technology Co, Ltd. (Shanghai, China).

### 2.2. Catalyst Preparation

A series of Mg-Zr composite oxide catalysts with different Mg/Zr ratio were prepared by hydrothermal process. Briefly, the typical preparation route could be described as follows: Mg (NO_3_)_2_·6H_2_O (0.64 g, 2.5 mmol) and ZrOCl_2_·8H_2_O (1.61 g, 5 mmol) were dissolved in the deionized water at room temperature, and the solution was added drop by drop to the 500 mL flask with the suitable amount of 2 mol/L NaOH solution at the same time by the co-current-precipitation process under vigorous stirring. Until the pH of the solution reached 11, and then stirred continuously for 30 min. Subsequently, the suspension was hydrothermally treated in a Teflon-lined stainless-steel autoclave at 150 °C for 6 h, and then calcined at 600 °C in flowing nitrogen atmosphere for 3 h. Depending on the molar ratio of Mg/Zr used in the preparation step, the catalysts were marked as Mg1Zr3, Mg1Zr2, Mg1Zr1, Mg2Zr1 and Mg3Zr1. Single metal oxide catalysts ZrO_2_ and MgO were synthesized in the same way as above composite oxide catalysts. The difference between co-precipitation process and hydrothermal process is that the mixture after stirring is allowed to stand at room temperature for 6 h without high-temperature treatment, and other steps remain unchanged. The catalyst samples prepared by hydrothermal method and coprecipitation method were named Mg_x_Zr_y_-HT and Mg_x_Zr_y_-CP respectively, where x/y was n(Mg)/n(Zr) molar ratio.

### 2.3. Characterization

The crystal phases of all samples were identified by powder X-ray diffractometer using D8 FOCUS (German Brook AXS Company, Karlsruhe, Germany) with Cu Kα radiation (40 kV) and a secondary beam graphite monochromator (SS/DS = 1°, RS 0.15 mm, counter SC). Talos F200s field emission transmission electron microscope (FEI company, Hillsboro, OR, USA) was used to observe the morphology and grain size of the catalysts. The strength and distribution of the basic/acid sites of the catalyst were determined by temperature programmed desorption of preadsorbed CO_2_ or NH_3_, which was performed using Auto Chem 2920 instrument. (Micromeritics, Norcross, GA, USA). The texture properties including the specific surface area, pore volume, and pore size of the catalysts were derived from N_2_ adsorption-desorption technique using 3H-2000PS2 (Beishied, Beijing, China) at −196 °C. The catalysts were pretreated by outgassing in vacuum at 200 °C for 3 h before measurement. X-ray photoelectron spectroscopy (XPS) data were collected on a Thermo Scientific K-Alpha electron spectrometer (Thermo Fisher, Waltham, MA, USA) equipped with Al Kα radiation (hv = 1486.6 eV).

### 2.4. Catalytic Activity Test

Transesterification of GL to GC was carried out in a round bottom flask with reflux condenser at atmospheric pressure. A total of 3.3 g of GL and 16.3 g of DMC were added into a 100 mL round-bottomed flask, the reaction mixture was heated to 90 °C while stirring in oil bath, then the catalyst of 3 wt% of GL was added to the reaction mixture. After the desired time, the products were separated by centrifugation and analyzed by gas chromatography using an Agilent 7890B gas chromatograph equipped with a DB-wax capillary column (30 m × 0.32 mm × 0.25 μm) and a hydrogen flame detector. The injector and detector temperatures were 250 °C and 300 °C, respectively. The yield of GC was calculated using internal standard method, in which N-butanol was the internal standard. The GL conversion, GC selectivity and yield were calculated by the following equations:

(1) $$\mathrm{GL}\;\mathrm{conversion}\left(\%\right)=\frac{\mathrm{mole}\;\mathrm{of}\;\mathrm{GL},\;\mathrm{feed}\;-\;\mathrm{mole}\;\mathrm{of}\;\mathrm{GL},\;\mathrm{final}}{\mathrm{mole}\;\mathrm{of}\;\mathrm{GL},\;\mathrm{feed}}\;\times \;100\;$$

(2) $$\mathrm{GC}\;\mathrm{selectivity}\left(\%\right)=\frac{\mathrm{mole}\;\mathrm{of}\;\mathrm{GC},\;\mathrm{produced}}{\mathrm{mole}\;\mathrm{of}\;\mathrm{GL},\;\mathrm{feed}-\mathrm{mole}\;\mathrm{of}\;\mathrm{GL},\;\mathrm{final}}\;\times \;100$$

(3) $$\mathrm{GC}\;\mathrm{yield}\left(\%\right)=\frac{\mathrm{GL}\;\mathrm{conversion}\;\times \;\mathrm{GC}\;\mathrm{selectivity}}{100}$$

## 3. Results and Discussion

### 3.1. Effect of Preparation Method

**Figure 1:**
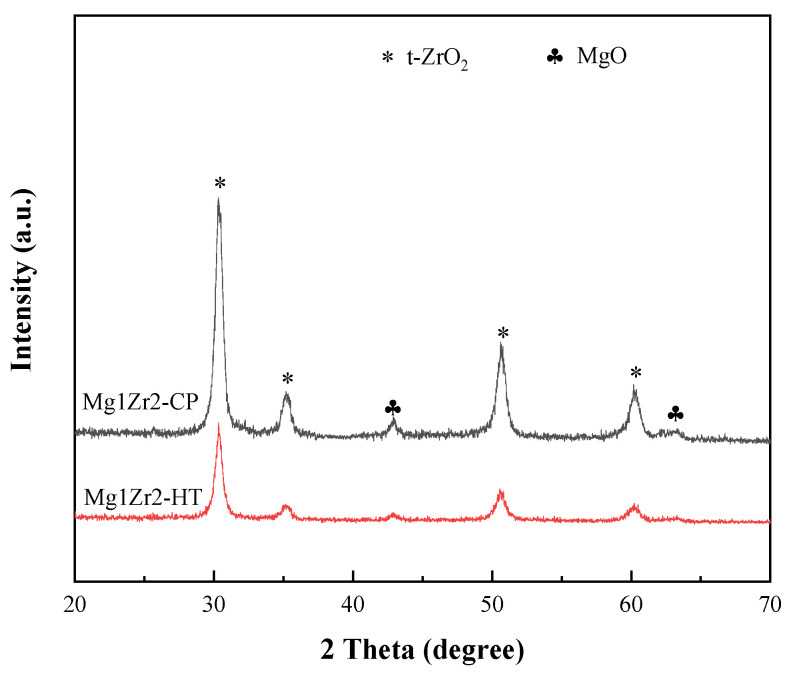
XRD patterns of Mg1Zr2-HT and Mg1Zr2-CP.

The XRD patterns of Mg1Zr2-HT catalyst prepared by hydrothermal process and Mg1Zr2-CP catalyst prepared by co-precipitation are shown in Figure 1. It can be seen from the figure that the diffraction peaks at 30.2°, 34.9°, 50.7° and 60.2° belong to tetragonal ZrO_2_ (t-ZrO_2_, JCPDS No. 50-1089), and there is no monoclinic ZrO_2_. t-ZrO_2_ has a unique bridging hydroxyl group and strong surface basicity, which is conducive to transesterification reaction [29]. Compared with ZrO_2_, the diffraction peak intensity of MgO is relatively weak, which is not due to the low content of Mg, but due to the low atomic scattering factor (atomic number) of Mg [30]. In addition, the grain sizes of Mg1Zr2-CP and Mg1Zr2-HT calculated by Scherrer formula are 13.4 nm and 13.1 nm respectively, and there is little difference between them.

The textural properties and surface basicity of Mg1Zr2-HT and Mg1Zr2-CP are summarized in Table 1. It can be seen that the Mg1Zr2-HT has a larger specific surface area and pore volume than Mg1Zr2-CP. This is because the intense collision between colloidal particles promotes the secondary pore formation of composite oxides under hydrothermal conditions, whereas the condensation between colloidal particles is a very slow process at room temperature. Therefore, the hydrothermal process is conducive to the formation of a more developed pore network structure, thereby improving the specific surface area and pore volume of Mg1Zr2-HT [31]. In addition, the dissolution deposition/crystallization process also occurs in the hydrothermal process [32]. Due to the dissolution of some precursors under hydrothermal conditions, the local solubility at the junction (neck) of the two colloidal particles will be lower than that at the nearby surface. Therefore, the deposition process will occur preferentially in the neck, resulting in the reinforcement of the colloidal network structure. During the subsequent calcination, the specific surface area and pore volume of the xerogel prepared by co-precipitation decrease rapidly due to the collapse of the gel skeleton and the sintering and growth of the catalyst particles [33].

The catalytic performance of the above two catalysts for GL transesterification was investigated, and the results are shown in Table 2. As can be seen from the data in Table 2, Mg1Zr2-HT and Mg1Zr2-CP both have good catalytic performance for GL transesterification, with GL conversion greater than 90% and GC selectivity of about 95%. Because Mg1Zr2-HT catalyst has a larger specific surface area, reactant molecules are more easily in contact with active sites, therefore have higher catalytic activity.

### 3.2. Effect of Mg-Zr Molar Ratio

**Figure 2:**
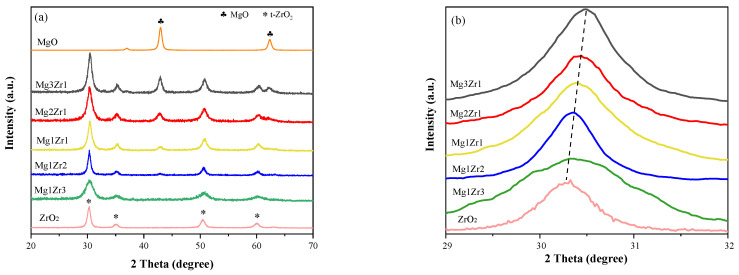
(**a**) XRD patterns of Mg-Zr composite oxides with different Mg/Zr ratios; (**b**) same as (**a**) but in region 2θ = 29–32.

The XRD patterns of catalysts with different Mg/Zr molar ratios are shown in Figure 2. It can be seen that with the increase of the Mg/Zr ratio, the diffraction peak of t-ZrO_2_ at 2θ of 30° gradually shifts to a high angle, which may be due to the doping of Mg^2+^ into the lattice of ZrO_2_, and some Zr^4+^ ions are replaced by Mg^2+^, resulting in the distortion of the crystal structure. Because the ion radius of Mg^2+^ is smaller than that of Zr^4+^ (the ion radius of Mg^2+^ and Zr^4+^ is 0.780 Å and 0.840 Å, respectively), the lattice shrinks, and the cell parameters decrease, so the corresponding 2θ shifts to high angle [34]. At a low Mg/Zr molar ratio, no diffraction peak of MgO is observed, indicating the formation of a solid solution. With the increase of Mg content, the characteristic diffraction peaks of periclase MgO (JCPDS No. 45-0946) were detected at 2θ of 43.2° (200) and 62.5° (220), and the intensity and sharpness gradually increased with the increase of Mg content, indicating that the particle size of MgO increased significantly. The lattice parameters and crystal plane spacing of Mg-Zr catalysts were analyzed by Jade, and the results are listed in Table 3. It was found that the lattice constant “a” and crystal plane spacing of (011) crystal plane of Mg-Zr catalyst decreased with the increase of Mg content, indicating that a stable and uniform Mg-Zr composite oxide structure was generated after the introduction of Mg^2+^ into t-ZrO_2_.

Figure S1a displays the Mg 1s spectra of Mg-Zr composite oxides catalysts, and the XPS spectrum of a single MgO is also presented for comparison. All the catalysts exhibited a broad and intense band centered at 1360 eV related to the emission from Mg 1s of Mg^2+^ in the oxide state. More importantly, the binding energies of Mg 1s in all the mixed oxides were lower than that of pure MgO, because the Mg-Zr oxides possessed a solid solution structure. The typical Zr 3d spectra are presented in Figure S1b. For pristine ZrO_2_, there appeared two peaks at 184.8 and 182.4 eV with a high intensity, which were associated with Zr 3d_3/2_ and Zr 3d_5/2_ energy states of Zr (IV) oxide species, respectively. The intensity of these two reflections gradually decreased with an increase in Mg content. Meanwhile, it is worth noting that adding Mg into ZrO_2_ support could give rise to a continuous increase of Zr 3d binding energy. These observations also support that Mg^2+^ had entered into the t-ZrO_2_ lattice, creating a solid solution. Dis-cussing the peak fitted O 1s spectra from Figure S1c. The peak at 531 eV, 533 eV, and 534 eV can be attributed to the presence of lattice oxygen species (O_L_), oxygen vacancies (O_V_), and chemisorbed oxygen species (O_C_). In general, the chemical valence of Zr ion is 4, but the Mg ion has only 2 valence, thus some vacancies are generated when substitution in order to keep charge neutrality in the ionic crystal, and these vacancies are favorable for heterogeneous catalysis [35]. It is worth noting that the Mg1Zr2 catalyst has the highest concentration of oxygen vacancies.

**Figure 3:**
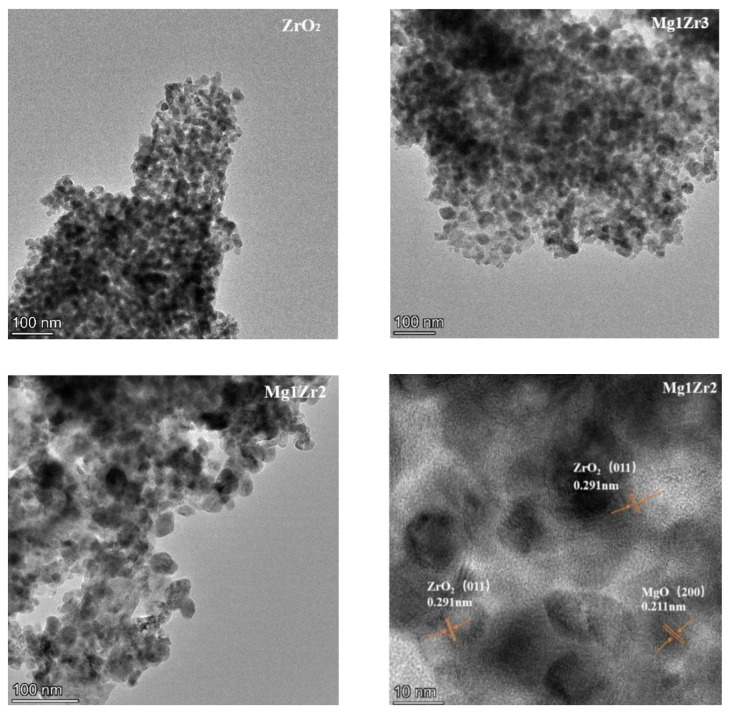

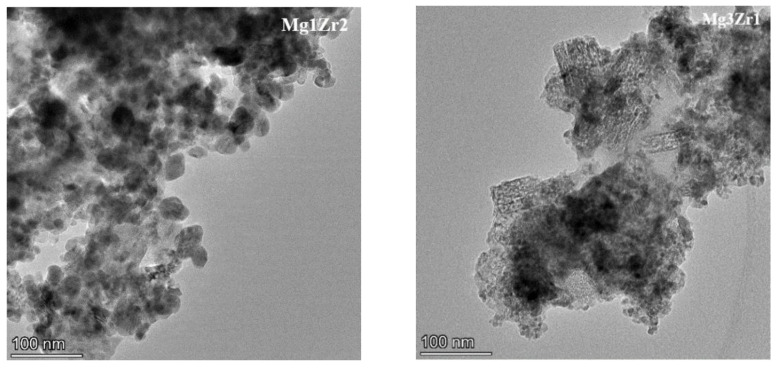
TEM images of Mg-Zr composite oxides with different Mg/Zr ratios.

In order to observe the microstructure and morphology of the catalyst, the Mg-Zr composite oxides with different Mg/Zr ratios were characterized by TEM, and the results are shown in Figure 3. It can be seen from the TEM image of ZrO_2_ that its particle size is relatively uniform, with an average particle size of 23 nm (based on the statistics of 91 particles in the TEM image), but its dispersion is poor, and a large number of particles agglomerate together. After adding a small amount of Mg, the uniformity of particle size of Mg1Zr3 becomes worse, indicating that the addition of Mg affects the crystallization and growth process of ZrO_2_. In addition, compared with ZrO_2_, there are some substances between the Mg1Zr3 particles, which may be extremely small MgO particles according to the preparation process and XRD results. With the increase of Mg content, the uniformity of ZrO_2_ particle size becomes worse, and the particles with a size of about 50 nm appear in Mg1Zr2. When Mg content exceeds Zr, ZrO_2_ particles gradually become smaller. Especially in Mg3Zr1, flake particle aggregates appear, and the large particle ZrO_2_ disappears completely. Sádaba et al. [30] prepared the Mg-Zr catalyst by co-precipitation method. They pointed out that in the preparation process, Zr^4+^ preferentially precipitated to form Zr(OH)_4_ or ZrO_2_(H_2_O)_X_. When most of Zr^4+^ was precipitated, Mg^2+^ formed Mg(OH)_2_ precipitation at pH 8~10. Therefore, Mg-Zr catalyst has an embedded structure with ZrO_2_ as core and MgO as a shell. According to the conclusion of Sádaba et al. [30] and TEM results, it can be inferred that MgO was formed in the outer layer of ZrO_2_ in the Mg-Zr catalysts prepared in this paper, which can be regarded as MgO wrapping ZrO_2_. Guan et al. [36] also believed that Mg^2+^ could enter the ZrO_2_ lattice to form Mg-Zr solid solution. When Mg content is large, MgO which cannot enter the lattice of ZrO_2_ can appear as an independent crystal phase and attach to the surface of magnesium-zirconium solid solution. The EDX spectrum and Elemental composition (Figure S2) show the presence of Mg and Zr. Even though several random areas were selected for the EDX test, the detected Mg/Zr molar ratio was almost the same as the theoretical value. The existence of all the elements in the oxide forms can be confirmed due to the presence of the high amount of oxygen and also the presence of Mg in Mg-Zr composite oxides enhances the basicity and stability of the catalyst.

**Figure 4:**
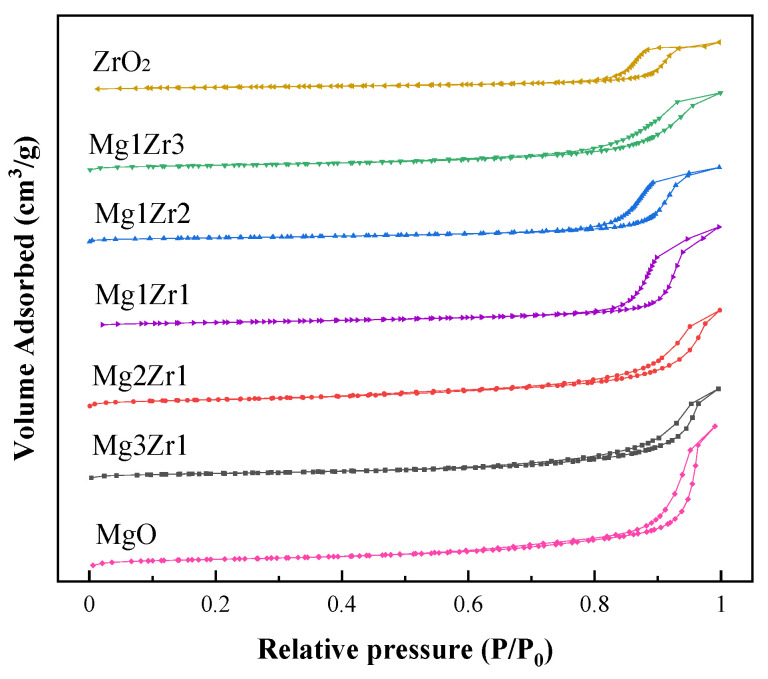
N_2_ adsorption-desorption isotherms of Mg-Zr oxides with different Mg/Zr ratios.

The N_2_ adsorption-desorption isotherms of Mg-Zr composite oxide catalysts are shown in Figure 4. There are obvious type IV adsorption equilibrium isotherms in the range of P/P_0_ = 0.5~1.0, indicating that the catalysts had mesoporous structures. ZrO_2_, Mg1Zr3, Mg1Zr2, and Mg1Zr1 catalysts all have H2 type hysteresis loops, indicating that the catalyst internal pore structure is ink bottle; The N_2_ adsorption-desorption isotherms of Mg2Zr1, Mg3Zr1, and MgO catalysts have no obvious saturated adsorption platform, accompanied by H3 hysteresis loop, indicating that the pore structure of the catalyst is very irregular, combined with the TEM results, it can be seen that there is the slit hole formed by the accumulation of flake particles.

The specific surface area, pore diameter, and pore volume of the catalysts are listed in Table 3. It can be seen that the specific surface area of Mg1Zr2 is 68.8 m^2^/g, and then the specific surface area increases with the addition of Mg, which is consistent with the experimental results of Guan [36]. As the Mg content increased, the specific surface area of Mg-Zr oxide catalysts has an upward trend, which may be due to the multi-layer dispersion of MgO attached to the surface of magnesium-zirconium solid solution, resulting in the increase of specific surface area. MgO has the largest specific surface area, but the catalytic performance is not the best, indicating that although the structure of the catalyst has a certain impact on the catalytic performance, it is not a completely decisive factor.

**Figure 5:**
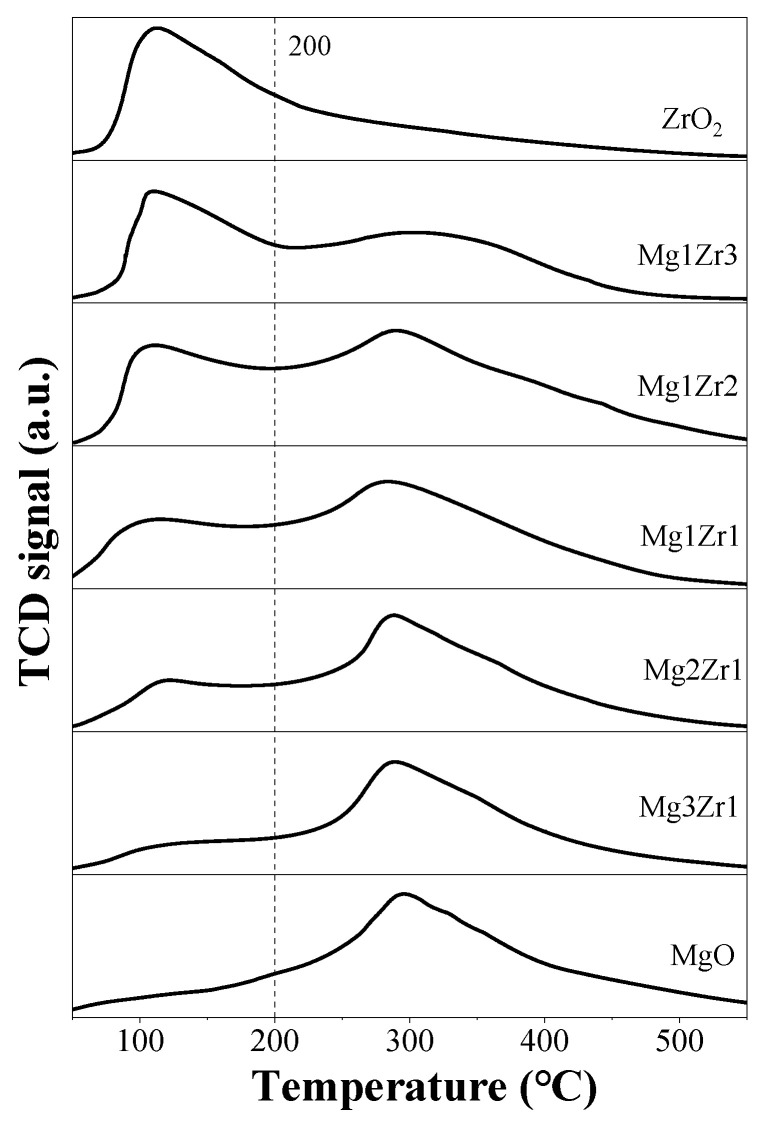
CO_2_-TPD of Mg-Zr composite oxides with different Mg/Zr ratios.

To better understand the intrinsic acid-base functionalities and correlate the catalysts with their catalytic behavior, CO_2_-TPD and NH_3_-TPD measurements were performed to quantitatively determine the distribution of surface acidity and basicity and the number of acidic and basic sites of MgO-ZrO_2_ catalysts. The CO_2_-TPD characterization of Mg-Zr oxide catalysts was carried out, and the influence of the Mg/Zr molar ratio on the basicity of the catalyst was investigated. The results are shown in Figure 5 and Table 3. It can be seen from Figure 5 that the Mg/Zr molar ratio has a significant effect on the basicity of Mg-Zr oxide catalysts. When the Mg content is 0 (ZrO_2_), there are mainly weak basic sites on the surface of the catalyst with a CO_2_ desorption temperature lower than 200 °C. With the addition of Mg, the number of medium strong basic sites (CO_2_ desorption temperature is in the range of 200–600 °C) on the catalyst surface gradually increases, while the number of weak basic sites decreases. Among them, Mg1Zr2 has the largest number of total basic sites, because it has more weak sites and medium and strong sites at the same time. With the further increase of Mg content, the number of weak basic sites decreased rapidly. The surface of the Mg3Zr1 catalyst is mainly composed of medium and strong basic sites, while the weak sites almost disappear. Its CO_2_-TPD curve is similar to that of MgO. The results showed that the weak basic sites on the surface of Mg-Zr oxide catalysts were mainly provided by ZrO_2_, while the medium and strong sites were mainly related to MgO. Zhang et al. [34] believed that the weak basic sites of the MgO-ZrO_2_ catalyst were related to its surface hydroxyl group, while the medium and strong basic sites were related to metal-oxygen pairs (Mg-O and Zr-O) and low coordination oxygen atoms (O^2−^). In addition, according to the data in Table 3, the total number of surface basic sites of ZrO_2_ and MgO is similar, but the number of Mg-Zr oxide catalysts increases significantly. In particular, the number of total basic sites of Mg1Zr2 catalyst reaches 145.3 μmol/g, which is 55.7% and 36.3% higher than that of the two single metal oxides, respectively. It is considered that Mg^2+^ and Zr^4+^ are fully mixed during the hydrothermal preparation of the catalyst, and part of Zr^4+^ in the lattice of ZrO_2_ is replaced by Mg^2+^ after calcination. Due to that Zr^4+^ is more positive than Mg^2+^, the electron density of O^2−^ in Mg-Zr oxide catalysts increases, thus increasing the number of medium and strong basic sites of the catalyst [28].

As shown in Figure S3, in the NH_3_-TPD curve of bare ZrO_2_, there are two NH_3_ desorption peaks at 130 °C and 530 °C, corresponding to weak acidic sites and strong acidic sites respectively. With the increase of MgO content, the medium-strength acid sites of the catalyst increased, while the weak and strong acid sites decreased. The results showed that there was no strong correlation between catalyst acidity and glycerol conversion. Although the role of acid sites in the activation of DMC cannot be completely ruled out, the effect of acid sites is less clear and predictable compared to the evident effect of basic sites [37].

**Figure 6:**
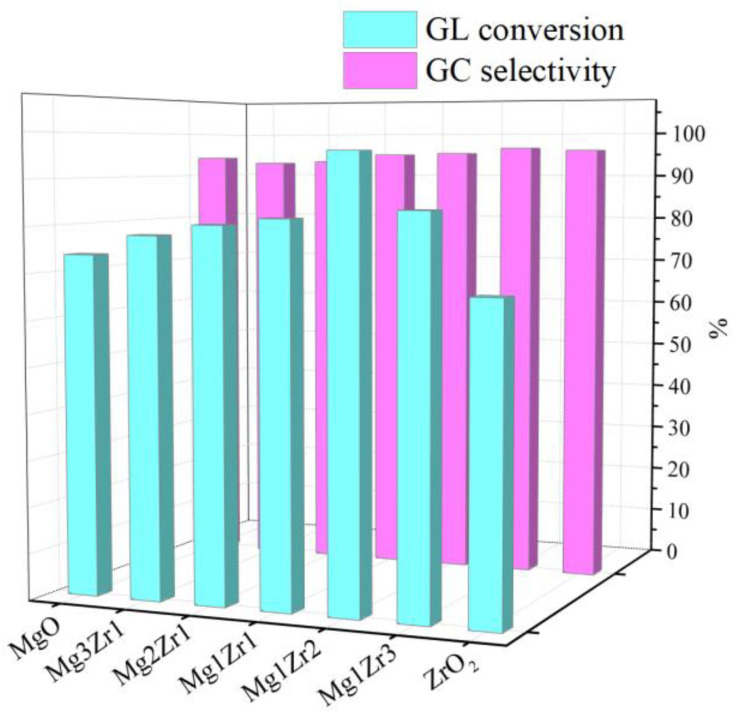
Catalytic performance of Mg-Zr oxides for transesterification of GL with DMC to GC (Reaction conditions: GL/DMC molar ratio = 1/5, catalyst loading = 3 wt% of GL, 90 °C, 60 min).

The effect of the Mg/Zr ratio on the transesterification of GL and DMC to GC over Mg-Zr composite oxide was studied, and the results are shown in Figure 6. As can be seen from the figure, ZrO_2_ and MgO alone are active for the transesterification of GL, and GL conversion is 67.2% and 73.8%, respectively. The activity of the Mg-Zr oxide catalysts was higher than that of the two single metal oxides, indicating the interaction between ZrO_2_ and MgO and improving the performance of the catalyst. When Mg1Zr3 was used, the GL conversion was 84.0%. With the increase of Mg content, the catalyst activity increased first and then decreased. Among them, Mg1Zr2 has the highest activity for transesterification of GL, with GL conversion of 96.0% and GC selectivity of 95.3%. The Mg/Zr ratio had little effect on the selectivity of GC. The byproduct was glycidyl, and no other products were detected. According to the characterization results, Mg1Zr2 has the highest number of total basic sites. Moreover, the order of GL conversion is basically the same as that of the number of basic sites on the catalyst surface. This indicates that the influence of the Mg/Zr ratio on catalyst performance lies in the change in the number of catalyst basic sites. In this transesterification reaction, the main function of the solid catalyst is to support the abstraction of H^+^ from glycerol by the basic sites so as to form glycerol anion. The higher the basicity of the catalyst, the more negative the charge of the glyceroxide anion (C_3_H_7_O_3_^−^), and consequently, the lower the free energy of the reaction [38]. In other words, the deprotonation of glycerol (on basic sites) is likely more important than the activation of dimethyl carbonate (on acidic sites) for the transesterification of glycerol and dimethyl carbonate [39].

### 3.3. Effect of Reaction Conditions on Transesterification of GL over Mg1Zr2-HT

Using Mg1Zr2-HT as a catalyst, the effects of reaction time, reaction temperature, catalyst amount, and GL/DMC molar ratio on the transesterification of GL with DMC to GC were investigated.

#### 3.3.1. Effect of Reaction Time

**Figure 7:**
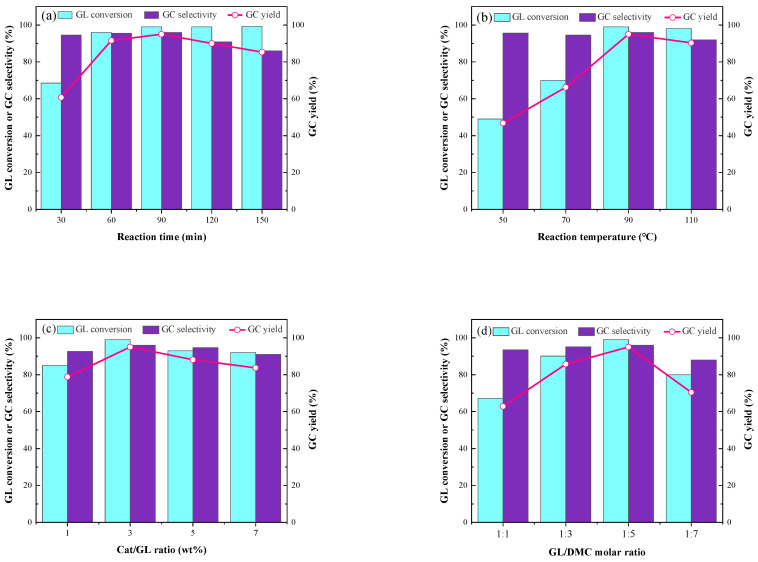
Effect of reaction conditions on the GL transesterification over Mg1Zr2-HT catalyst: (**a**) effect of reaction time (GL/DMC molar ratio = 1/5, catalyst amount = 3 wt% of GL, 90 °C), (**b**) effect of reaction temperature(GL/DMC molar ratio = 1/5, catalyst amount = 3 wt% of GL, 90 min), (**c**) effect of catalyst amount(GL/DMC molar ratio = 1/5, 90 °C, 90 min), (**d**) effect of GL/DMC molar ratio(catalyst amount = 3 wt% of GL, 90 °C, 90 min).

As shown in Figure 7a, the effect of reaction time on the transesterification of GL with DMC was investigated. It can be seen that GL conversion increased gradually with the increase in reaction time. When the reaction time was 90 min, the GL conversion was 99.0% and GC selectivity was 96.1%; With the continuous extension of reaction time, GL conversion remained unchanged and GC selectivity decreased. This was caused by the decomposition of GC into glycidyl.

#### 3.3.2. Effect of Reaction Temperature

It can be seen from Figure 7b that increasing temperature before 90 °C is conducive to promoting the reaction. This is because the reaction equilibrium constant of this reaction increases with the increase of temperature, so heating is conducive to the reaction. At 90 °C, GL conversion was 99.0% with GC selectivity of 96.1%. When the temperature continues to rise, the decomposition of GC into glycidyl occurs more readily [40], so GC selectivity decreases.

#### 3.3.3. Effect of Catalyst Amount

The transesterification reaction of glycerol was highly influenced by the catalyst amount (wt% based on GL) and presented in Figure 7c, the increase of the amount of catalyst from 1 wt% to 3 wt%, the GL conversion and GC yield gradually increased, which was attributed to the increase in the basic sites of the transesterification catalyst. However, the amount of catalyst increased from 3 wt% to 7 wt%, and the GC yield decreased slowly, which may be due to the agglomeration of catalyst at a higher amount, which makes the reactants unable to enter the active center of the catalyst. The higher the amount of catalyst is, the greater the mass transfer resistance is, which may hinder the transesterification of GL with DMC [41].

#### 3.3.4. Effect of the Molar Ratio of GL/DMC

The molar ratio of GL/DMC has a great influence on the GL conversion and GC yield during the transesterification. Since the transesterification reaction is essentially reversible, excessive DMC is needed to shift the reaction equilibrium to GC. From Figure 7d, it is clear that with the increase of the molar ratio of DMC/GL, the conversion of GL showed an upward trend, and when the molar ratio was 1/5 (GL/DMC), the maximum conversion was 99.0% and the GC selectivity was 96.1%. If the molar ratio of DMC/GL continues to increase, the conversion of GL and GC yield decreases. This may be due to the excessive DMC diluting the catalyst and limiting the contact between GL and the catalyst, thus reducing the reaction rate [40].

### 3.4. Catalyst Stability

**Figure 8:**
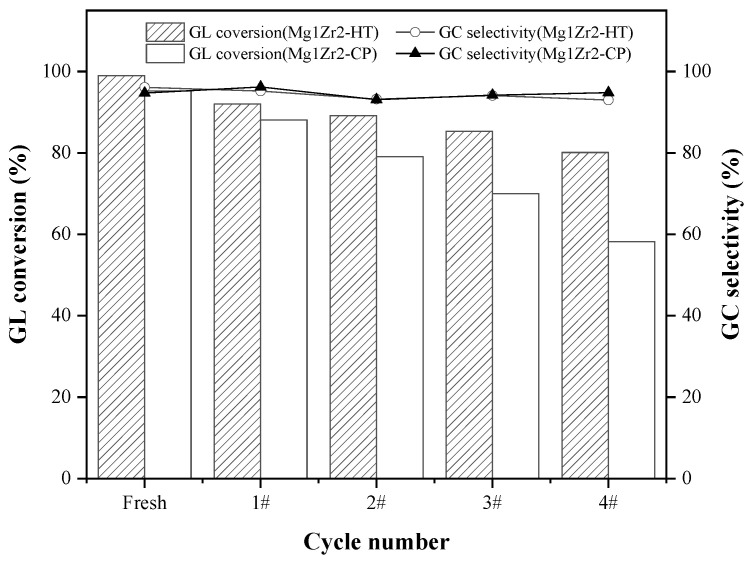
Reusability of Mg1Zr2-HT and Mg1Zr2-CP (Reaction condition: GL/DMC molar ratio = 1/5, catalyst loading = 3 wt%, 90 °C, 90 min).

The reusability of a catalyst is an important index to evaluate the performance of the catalyst. In this study, the reusability of Mg1Zr2-HT and Mg1Zr2-CP catalysts for transesterification of GL with DMC is compared, as shown in Figure 8. After the reaction, the catalyst was centrifuged, washed three times with methanol, dried at 100 °C, and then calcined at 600 °C in air for 3 h. As can be seen from the figure, GC selectivity was little affected by repeated use and was almost constant. However, GL conversion gradually decreased, and there were significant differences between the Mg1Zr2-HT and Mg1Zr2-CP catalysts. When a fresh catalyst was used, the GL conversion over Mg1Zr2-HT and Mg1Zr2-CP catalysts was 99.0% and 95.2%, respectively. Moreover, when repeated for the fourth time, GL conversions were 80.1% and 58.2%, respectively. The stability of Mg1Zr2-HT is much better than that of Mg1Zr2-CP.

In order to explore the reasons for the differences between the two catalysts, Mg1Zr2-HT and Mg1Zr2-CP catalysts after four times of reused were characterized by XRD, N_2_ adsorption-desorption, CO_2_-TPD, TEM, and XPS.

**Figure 9:**
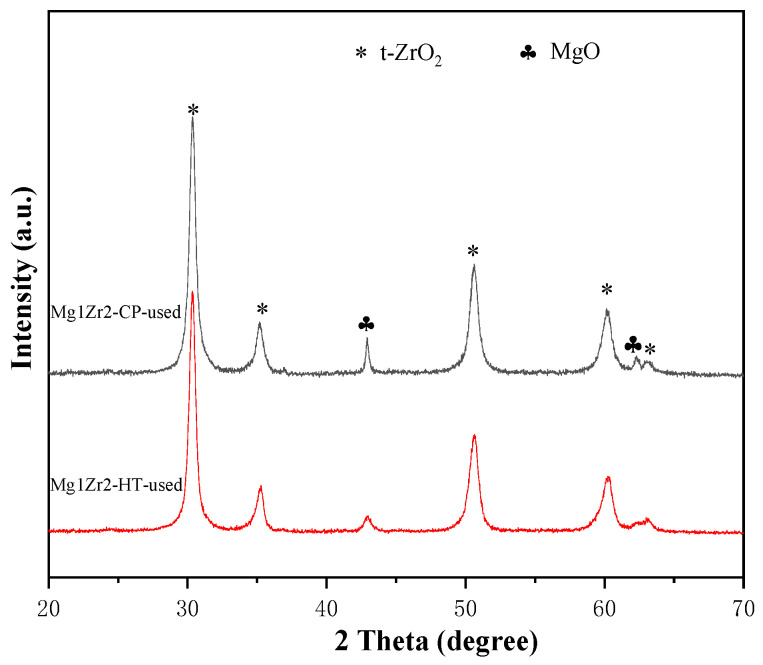
XRD patterns of Mg1Zr2-HT-used and Mg1Zr2-CP-used.

The XRD patterns of Mg1Zr2-HT-used and Mg1Zr2-CP-used catalysts after the fourth cycle are presented in Figure 9. It can be seen that there are obvious characteristic diffraction peaks at 2θ of 30.2°, 34.8°, 50.7°, 60.2°, and 62.9°, corresponding to (011), (110), (020), (121) and (202) crystal planes of tetragonal ZrO_2_, respectively. The characteristic diffraction peaks appear at 2θ of 43.2° and 62.5°, corresponding to the (200) and (220) crystal planes of MgO, respectively. Based on the diffraction peaks of ZrO_2_ (011) and MgO (200) crystal planes, the grain sizes of ZrO_2_ and MgO in Mg1Zr2-HT-used are 15.8 nm and 15.0 nm by the Scherrer formula, respectively; The grain sizes of ZrO_2_ and MgO in Mg1Zr2-CP-used are 16.6 nm and 31.4 nm, respectively. Compared with the fresh catalyst, the particle sizes of the two catalysts after repeated use both increased, but the grain size of MgO in Mg1Zr2-CP-used increased by about double, while the grain size of MgO in Mg1Zr2-HT-used increased by only 14%. Under hydrothermal conditions, ions in the solution automatically aggregate to form the most stable chemical structure that cannot be decomposed in the system during the temperature change, so they have good grain stability.

**Figure 10:**
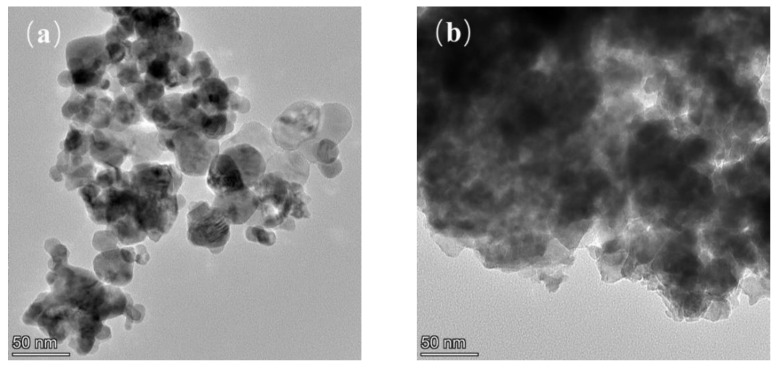
TEM images of Mg1Zr2-HT-used (**a**) and Mg1Zr2-CP-used (**b**).

It can be seen from Figure 10 that after transesterification, the particle sizes of both two catalysts increased significantly and the particle sizes became uneven, indicating that the catalyst particles appeared aggregate and sintering, resulting in the gradual increase of grain size and the decrease of dispersion. The Mg1Zr2-CP-used catalyst had serious agglomeration, while the Mg1Zr2-HT-used catalyst had slight sintering but no obvious agglomeration, indicating that the catalyst prepared by the hydrothermal process had strong sintering resistance. This is because under hydrothermal conditions, the compounds in the solution may renucleate and restructure, so that the particles after hydrothermal treatment have better dispersion and grain stability than those particles only by neutralization precipitation [42].

Compares the deconvoluted Mg 1s, Zr 3d, and O 1s XPS spectra of fresh and used Mg1Zr2-HT. The relative abundances of the Mg 1s, Zr 3d, and O 1s of the samples from Figure S4 showed that the content of the oxygen vacancy decreased from 26.5% to 23.1% and the content of the chemisorbed oxygen species increased from 1.1% to 3.9%, respectively, indicating that irreversible deactivation was caused.

As can be seen from Table 4 that the Mg1Zr2-HT-used catalyst has a larger specific surface area than the Mg1Zr2-CP-used catalyst, and more active sites can be retained. This may be because the colloidal network structure formed in the hydrothermal process is more stable through dissolution-deposition, which alleviates the collapse of the structure and the sintering of particles, so that it still maintains a large specific surface area in the reaction process [43]. Since the catalytic reaction occurs on the surface of active components, the agglomeration and growth of grains lead to the decrease of active surface area, the decrease of active sites, and the reduction in catalytic activity [44]. Compared with fresh catalysts, the number of weak basic sites of Mg1Zr2-HT and Mg1Zr2-CP catalysts decreased by 12% and 13%, respectively, but the number of medium and strong basic sites decreased by 25% and 50%, respectively. Moreover, it was observed that Mg1Zr2-CP-used suffered a greater loss of basic sites than Mg1Zr2-HT-used. This is probably the reason why Mg1Zr2-HT showed better catalytic performance than Mg1Zr2-CP after four cycles. Mg1Zr2-HT possesses a more stable crystal structure to avoid the irreversible reduction of basic sites amount.

## 4. Conclusions

In this work, Mg-Zr composite oxide catalysts with different Mg/Zr molar ratios were prepared by hydrothermal process and their activity and stability towards GC synthesis were studied. The results showed that the catalysts prepared by the hydrothermal process had larger specific surface area, smaller grain size, and higher dispersion than those prepared by the co-precipitation process. The Mg1Zr2-HT catalyst calcined at 600 °C in a nitrogen atmosphere showed the best catalytic performance, with GL conversion of 99% and GC selectivity of 96.1% under mild reaction conditions. This is attributed to the balanced strong and weak basic sites and highly dispersed MgO. Moreover, the GL conversion was demonstrated to increase in parallel with the total amount of basic sites. Compared with the Mg1Zr2-CP catalyst, the Mg1Zr2-HT catalyst has good thermal stability and reproducibility. The conversion of GL is still up to 80.1% and the selectivity of GC is 93.0% in the fourth reuse, while the regenerated Mg1Zr2-CP catalyst is 58.2% and 94.8% in the fourth reuse. The reason for the difference may be that in the cyclic reaction process, Mg1Zr2-HT has good grain stability and small growth amplitude, but the grain growth of active species in Mg1Zr2-CP is large, which will greatly reduce the effective active surface area of the catalyst, resulting in a significant decrease in the catalytic performance.

## Figures and Tables

**Table 1:** Texture properties and surface basicity of Mg1Zr2-HT and Mg1Zr2-CP.

Catalyst	ZrO_2_ Crystallite Size ^a^ (nm)	MgO Crystallite Size ^b^ (nm)	S_BET_ ^c^ (m^2^/g)	D_P_ ^d^ (nm)	V_P_ ^e^ (cm^3^/g)	Basicity ^f^ (μmol/g)		
						W	M + S	Total
Mg1Zr2-HT	13.1	13.2	68.8	24.0	0.41	57.1	88.2	145.3
Mg1Zr2-CP	13.4	14.9	42.8	26.9	0.28	53.2	82.6	135.8

^a^ Calculated by Scherrer formula using the full width at half maximum of ZrO_2_ (011) plane. ^b^ Calculated by Scherrer formula using the full width at half maximum of MgO (200) plane. ^c^ S_BET_ was measured by the multi-point BET method. ^d^ Average pore size was calculated from the desorption branch of the isotherm using the BJH method. ^e^ Total pore volume was measured at P/P_0_ = 0.99. ^f^ Calculated by the results of CO_2_-TPD.

**Table 2:** Catalytic performance of Mg1Zr2-HT and Mg1Zr2-CP for transesterification of GL with DMC.

Catalyst	GL Conversion (%)	GC Selectivity (%)	GC Yield (%)
Mg1Zr2-HT	96.0	95.1	91.3
Mg1Zr2-CP	91.0	94.0	85.5

Reaction condition: GL/DMC molar ratio = 1/5, catalyst loading = 3 wt% of GL, 90 °C, 60 min.

**Table 3:** Texture properties and surface basicity of various Mg-Zr composite oxides.

Catalyst	ZrO_2_ (011)		Lattice Parameter (nm)		Particle Size ^a^ (nm)	S_BET_ ^b^ (m^2^/g)	Dp ^c^ (nm)	Vp ^d^ (cm^3^/g)	Basicity ^e^ (μmol/g)		
	2θ (°)	d (nm)	a	c					W	M + S	Total
ZrO_2_	30.25	0.2952	0.3622	0.5112	12.3	46.4	22.9	0.27	72.9	20.4	93.3
Mg1Zr3	30.30	0.2947	0.3601	0.5097	9.6	86.8	19.5	0.42	65.3	64.1	129.4
Mg1Zr2	30.33	0.2945	0.3596	0.5116	13.1	68.8	24.0	0.41	57.1	88.2	145.3
Mg1Zr1	30.36	0.2942	0.3594	0.5119	8.0	78.5	27.0	0.46	38.6	86.1	124.7
Mg2Zr1	30.38	0.2940	0.3593	0.5102	7.6	87.0	22.5	0.49	31.2	89.3	118.5
Mg3Zr1	30.45	0.2933	0.3586	0.5106	9.7	112.0	18.3	0.52	19.8	92.6	112.4
MgO	-	-	-	-	-	114.0	27.5	0.78	10.5	96.1	106.6

^a^ Calculated by Scherrer formula using the full width at half maximum of ZrO_2_ (011) plane. ^b^ S_BET_ was measured by the multi-point BET method. ^c^ Average pore size was calculated from the desorption branch of the isotherm using the BJH method. ^d^ Total pore volume was measured at P/P_0_ = 0.99. ^e^ Calculated by the results of CO_2_-TPD.

**Table 4:** Texture properties and surface basicity of Mg1Zr2-HT-used and Mg1Zr2-CP-used.

Catalyst	ZrO_2_ Crystallite Size ^a^ (nm)	MgO Crystallite Size ^b^ (nm)	S_BET_ ^c^ (m^2^/g)	D_P_ ^d^ (nm)	V_P_ ^e^ (cm^3^/g)	Basicity ^f^ (μmol/g)		
						W	M + S	Total
Mg1Zr2-HT-used	15.8	15.0	40.1	30.3	0.30	50.3	66.2	116.5
Mg1Zr2-CP-used	16.6	31.4	25.5	29.7	0.19	46.2	42.0	88.2

^a^ Calculated by Scherrer formula using the full width at half maximum of ZrO_2_ (011) plane. ^b^ Calculated by Scherrer formula using the full width at half maximum of MgO (200) plane. ^c^ S_BET_ was measured by the multi-point BET method. ^d^ Average pore size was calculated from the desorption branch of the isotherm using the BJH method. ^e^ Total pore volume was measured at P/P_0_ = 0.99. ^f^ Calculated by the results of CO_2_-TPD.

## Data Availability

The data presented in this study are available on request from the corresponding author.

## Supplementary Materials

The following supporting information can be downloaded at: www.mdpi.com/article/10.3390/nano12121972/s1, Figure S1: X-ray photoelectron spectra of Mg-Zr composite oxides. Regions: Mg 1s (a), Zr 3d (b) and O 1s (c); Figure S2: EDX spectrum and Elemental composition of Mg-Zr composite oxides with different Mg/Zr ratio; Figure S3: NH_3_-TPD of Mg-Zr composite oxides with different Mg/Zr ratio; Figure S4: X-ray photoelectron spectra of fresh and used Mg1Zr2 composite oxides. Regions: Mg 1s (a), Zr 3d (b) and O 1s (c).
