# Supplementary material for: Transesterification of Glycerol to Glycerol Carbonate over Mg-Zr Composite Oxide Prepared by Hydrothermal Process
Source: Nanomaterials (Basel). 2022 Jun 8;12(12):1972. doi: 10.3390/nano12121972 (PMC9227028; doi:10.3390/nano12121972)
Supplement: Supplementary file 1 [file nanomaterials-12-01972-s001.zip › nanomaterials-1721669-supplementary.pdf]

nanomaterials

MDPI

Supplementary Materials

Transesterification of glycerol to glycerol carbonate over Mg-Zr composite oxide prepared by hydrothermal process

Yihao Li 1, Hepan Zhao 1, Wei Xue 1, Fang Li 1, * and Zhimiao Wang 1, *

[1] Key Laboratory of Green Chemical Technology and High Efficient Energy Saving of Hebei Province, Tianjin Key Laboratory of Chemical Process Safety, School of Chemical Engineering, Hebei University of Technology, Tianjin 300401, China; 201921503002@stu.hebut.edu.cn; hgdzhao16029@hebut.edu.cn; weixue@hebut.edu.cn

* Correspondence: lifang@hebut.edu.cn; wangzhimiao@hebut.edu.cn

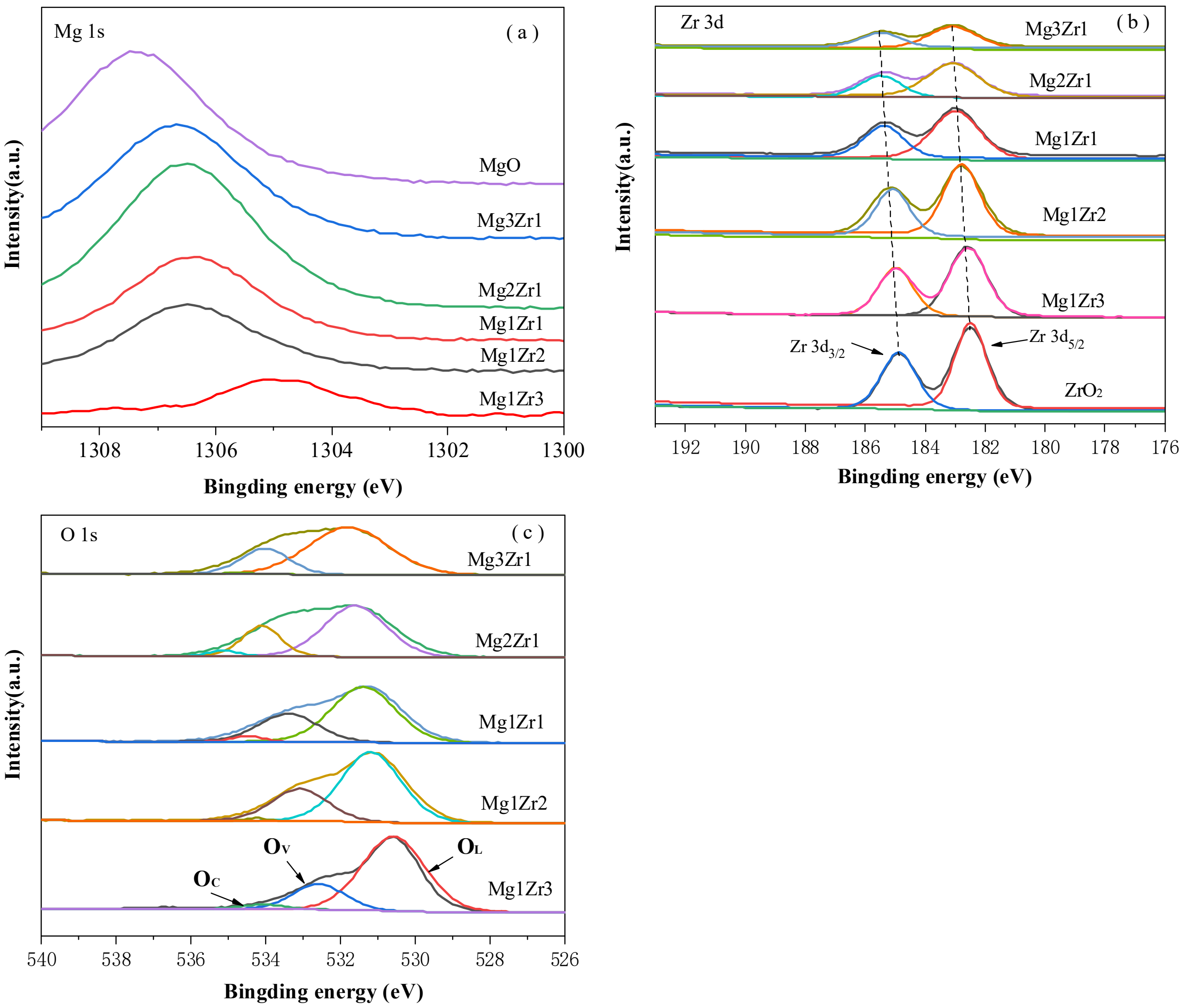

Figure S1. X-ray photoelectron spectra of Mg-Zr composite oxides. Regions: Mg 1s (a), Zr 3d (b) and O 1s (c).

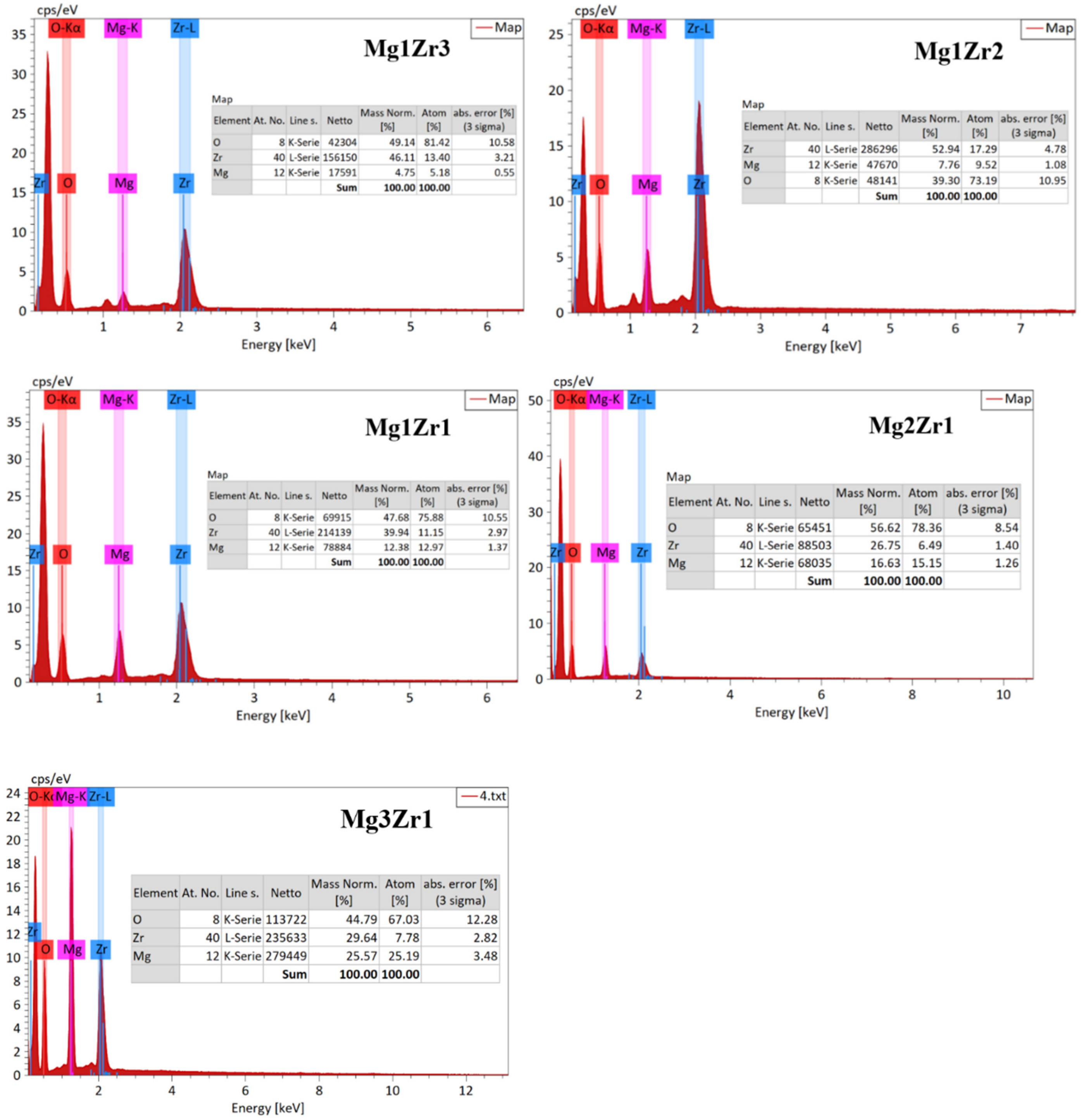

Figure S2. EDX spectrum and Elemental composition of Mg-Zr composite oxides with different Mg/Zr ratio.

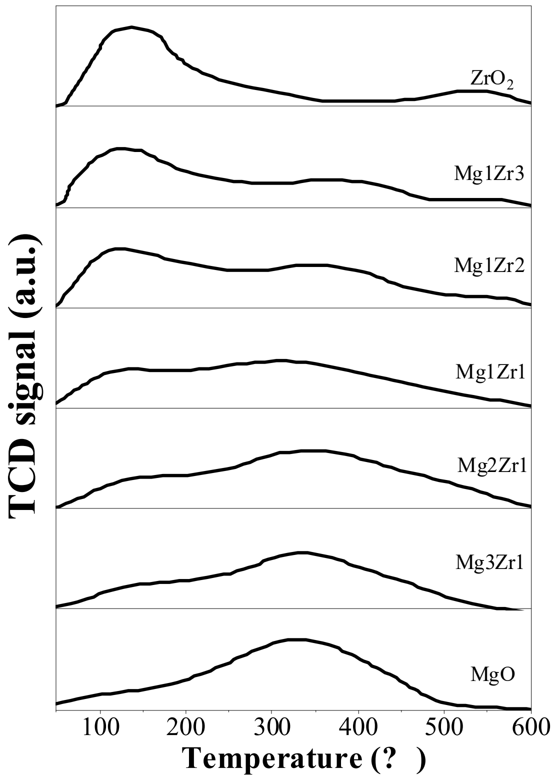

Figure S3. NH_3-TPD of Mg-Zr composite oxides with different Mg/Zr ratio.

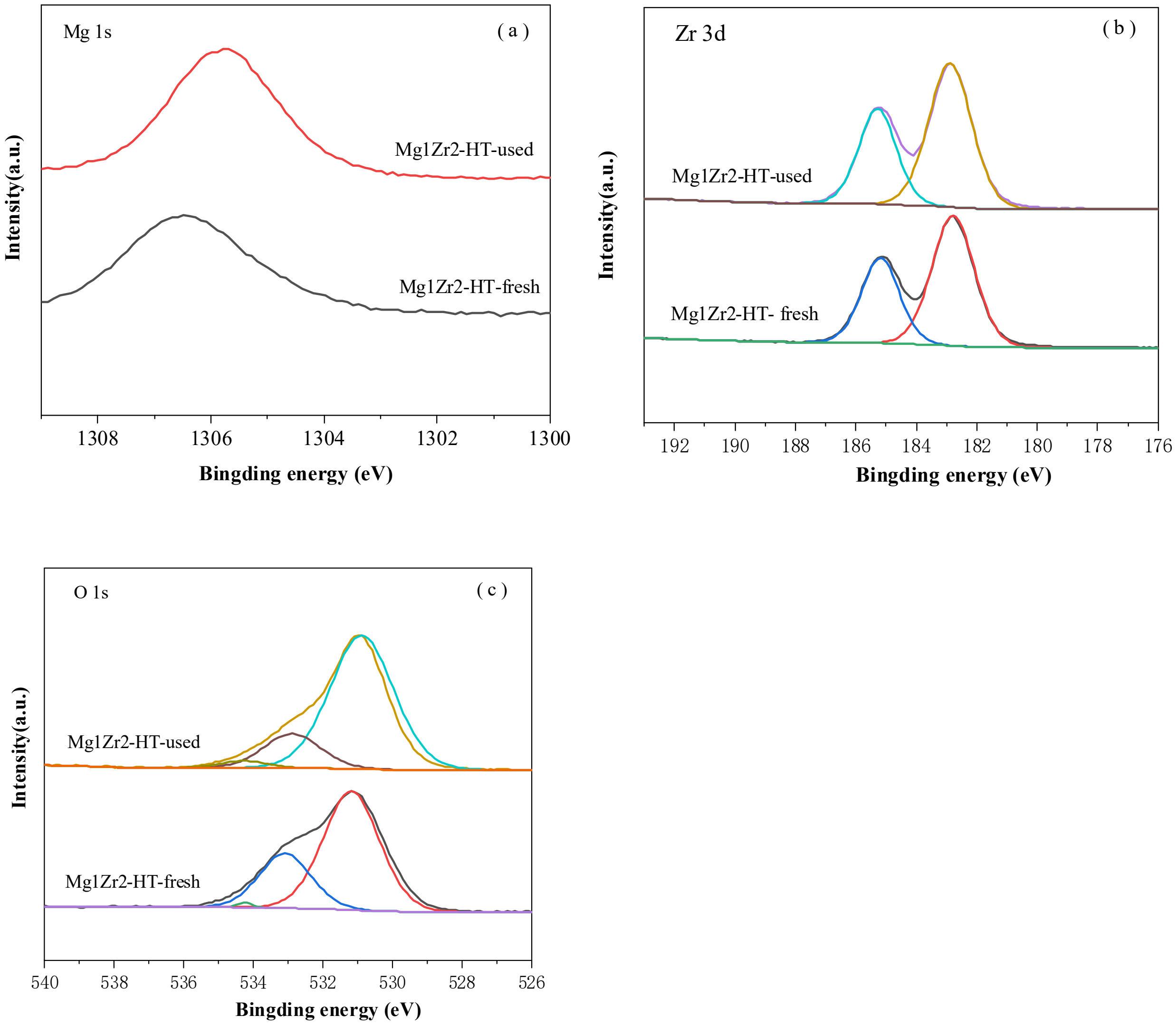

Figure S4. X-ray photoelectron spectra of fresh and used Mg1Zr2 composite oxides. Regions: Mg 1s (a), Zr 3d (b) and O 1s (c).
